# Culture intelligent workflow, structure, and steps

**DOI:** 10.3389/frai.2023.985469

**Published:** 2023-02-28

**Authors:** James Andrew Henry

**Affiliations:** ^1^Institute of Biomedical Sciences, London, United Kingdom; ^2^Society for Advanced Blood Management, Mount Royal, NJ, United States; ^3^British Blood Transfusion Society, Birmingham, United Kingdom

**Keywords:** population, health, management, artificial, intelligence

## Abstract

**Introduction:**

Technologies abstract intelligence and provide predictor and precision insight in workflows that manage disorders, similar to cardiology and hematological disease. Positive perceptions of Artificial Intelligence (AI) that support Machine Learning (ML) and Deep Learning (DL) manage transformations with a safe system that improves wellbeing. In sections, workflow introduces an eXamination (X = AI) as an end-to-end structure to culture workstreams in a step-by-step design to manage populace health in a governed system.

**Method:**

To better healthcare outcomes, communities and personnel benefit from an explanation and an interpretive that elucidates workflow for citizens or practitioners to comprehend personalized platforms. Therefore, the author undertook structure and practice reviews and appraised perspectives that impact the management of AI in public health and medicine.

**Results:**

Figures for the management of AI workflow illustrate and inform on the model, structure, culture, assurance, process steps, values, and governance required for abstract insights in public health and medicine. The papers' end-to-end structure with explanans in a work culture interprets the step-by-step designs that manage the success of AI. Personalized care graphics offer an explanandum in the management of biological analytic value.

**Discussion:**

Healthcare leadership collaboratives plan population health with an upstream, workplace and workstream format. Secure workflow and safety wellbeing system requirements prove that genomics and AI improve medicine. Therefore, the paper discusses group understanding of current practice, ethics, policy, and legality.

**Conclusion:**

“Culture, intelligent workflow, structure, and steps” improve wellbeing with personalized care and align a percept for national opportunities, regional control, and local needs. Personalized practice cultures support analytic systems to describe, predict, precision, and prescript medicine in population health management eXaminations.

## Workflow introduction

An intro to workflow provides personalized solutions for global health management. Background to the eXamination concept in medical examinations develops AI across all healthcare sector operations in a series of papers that govern through international standard developments. The system prelude and the paper provide an explanation and interpretive of AI while elucidating future insights into Population Health Management for opinions.

### System prelude

**Figure 1:**
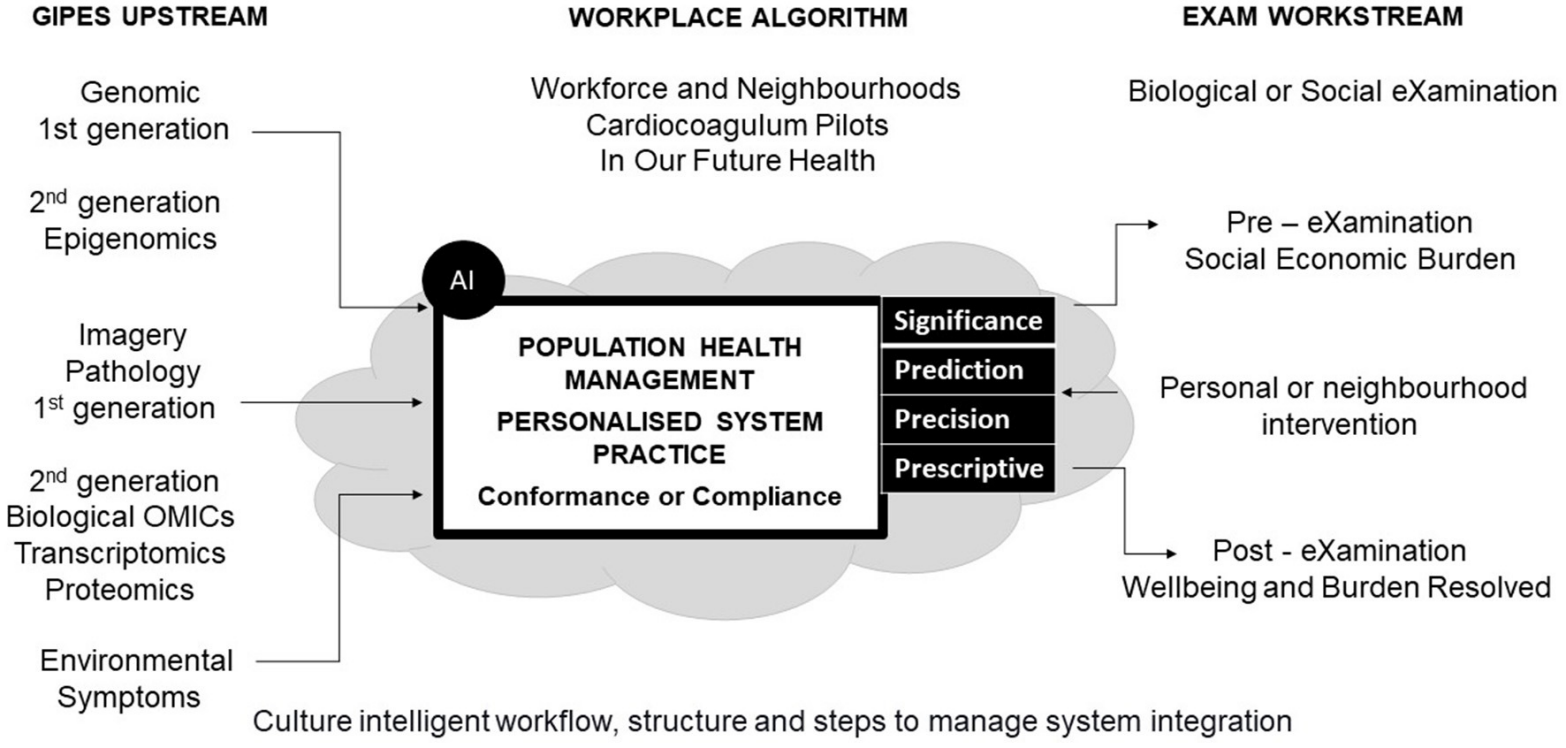
Model workflow.

AI projects and programs like cardio with coagulum, patient blood management, elderly comorbid care, and newborn screening benefit from Population Health Management. As illustrated throughout the manuscript, workflow delivers upstream data for workplace ML and DL eXaminations that workstream medical solutions (Figure 1). Real-time or batch quality data for insight delivered in practice with healthcare AI governance have ML and DL in the eXamination process and its safety management system. Personalized system practice evolves in a series of papers titled; -

1. Atrial Fibrillation the Genomics Mission
2. Culture Intelligent Workflow, Structure, and Steps
3. Population Health Management Standard eXaminations
4. Personalized System Practice and Group Determinants
5. Personalized System Practice—eXamination Transformation
6. Personalized System Practice—eXamination Improvement

### Global health management

The World Health Organization supports data-driven services in healthcare for “early detection, diagnosis, and medical decision making,” as the future of Population Health Management (PHM) engages practitioner and citizen users on a global scale (Wiegand et al., 2019). The US introduced the Genomics and Personalized Medicine Act, which called for the study of barriers to implementing personalized medicine, impacting government rethinks on AI (Margetts and Dorobantu, 2019).

A forward-thinking Australian federal state with public funds integrated a flexible and adaptable genomic system-wide approach to medicine. Whilst successful, the lessons learned included budgeting reviews and, most notably, insight on oversight (Vidgen et al., 2021).

The UK Health and Care Bill empowers the secretary of state for health and social care to capitalize on digital and genomic innovations and engage the British Medical Association with digital Life Sciences in PHM (Iacobucci, 2021).

Governments build back better with value and principles to integrate datasets on digital channels, wherein oversight on workflow co-ordinates a central role for diagnostics with informatic core purpose to reduce socio-economic burdens (Minkman, 2016; Boehme et al., 2021).

If not remedy for other countries, the UK resolves variance with a public health approach to workflow with a digital guide to “What Good Looks Like” (Public Health England, 2019). Principles in action seek to enable workflow eXaminations to become the norm to predict health and precision care (Public Health England, 2019).

Globally, responsible genomic data sharing for human health benefits requires not only policy-framing but setting of technical standards (Knoppers, 2014). The UK Multi-Agency Advice Service provides a regulatory collaborative approach for developer or adopter of phenomics to deliver a life sciences vision for clinical research and genomics at scale for wellbeing [International Organization for Standardization (ISO), 2018a]. International workflows organize to sustain system biology success in practice, as digital maturity commences with a standard for self-assessment (NHS, 2022a). PHM at the point of care executes workflow competence through a system standard common to genomics, image, and pathology laboratories [International Organization for Standardization (ISO), 2012]. To build a digital data strategy with intelligence norms integrate upstream, workplace and workstreams to realize personalized biological eXaminations that impact socioeconomic success [International Organization for Standardization (ISO), 2017, Figure 1].

### Workflow background

In 2014, the Barnes report recommended that pathology quality systems integrate hospital trust governance to reduce variable adversities (Pathology Quality Assurance Review [Internet], 2014), similar to cardiology or hematology diagnostics that impact clinical decisions. In 2017, the author presented an abstract at a UK national assurance conference for a Patient Blood Management Quality System proposal which summarized the need for life science intercepts (Henry, 2017).

In 2019, the US Society for Advanced Blood Management presented an abstract on an atrial fibrillation (cardio) related coagulation (coagulum) in genomic futures, as the author framed cardiocoagulum champions (Henry, 2019). Further analysis of cardiocoagulum socioeconomic risks, predictive health, and precision care opportune a genomic mission for our industrial lives with workflow for PHM in Future Health (Our Future Health, 2021).

Today, the UK genomic strategy for end-to-end delivery of research, preventative and precision pillars build on data-driven themes (GENOME UK, 2020). Digital AI strategy support regional analytic study designs for long-term citizen needs (GOV.UK, 2021a), with workplace and homeplace delivery proposals for personalized eXaminations.

In moving forward, Integrated Care Systems (ICS) bring medical debate on AI clinical studies that secure data flow for safe PHM (Shelmerdine et al., 2021). Citizens' needs, assessed with genomics, build model ontology foundations in an end-to-end structure that engage and operate for communities as ICS boards precision medicine (Aronson and Rehm, 2015).

## End-to-end structure

Workflow designs shows health provider or communes that populace health can be managed with upstream features for workplace intelligence that workstream eXaminations which intercept biological and socio-economic burdens (Figure 1). Shaping digital futures on Biological Ontology Models across a Model Hospital that includes social care requires workflow comprehension and function to get the best out of AI (Hardie et al., 2021; Maguire et al., 2021). Planning with our communities and presenting algorithm assurance enhance health cultures as alliances bring end-to-end structures with delivery of core eXaminations for accurate support decisions (Department of Health and Social Care Public Health England, 2020; NHS England, 2020a).

### Upstream features

Predictive health and precision care reduce the frequency of harm to wellbeing with an upstream of biological data, similar to features for Genomic variations, tissue Images, and Pathology diagnostics [GIP] data. Cardiocoagulum pre-eXamination inputs nucleotide informatics, with or without other informatics, to personalize health with a population health management requirement (Table 1).

**Table 1:** Cardiocoagulum table.

**Workflow upstream features to personalize and**		
**precision medicine**		
Genomic domain variations	Pathology or image data	Predict health or precise care
Poly gene scores	ECG trace	Atrial fibrillation, heart failure
Single nucleotide polymorph	TTE and TTO image	Stroke, CVD, dementia
Insertion or deletion	MRI image	Hyperlipidemia, hypertension
Exomiser SNV	Viscoelastic graphics	Coagulation disorder, bleed
Exomiser InDel	Platelet function	Thrombocytopenia, thrombus
Nucleotide copy number	RBC and platelet image	Anemia, polycythemia
Short tandem repeat	Proteomics trace	Multi purposes

SNV, single nucleotide variants; ECG, electrocardiograph; CVD, cardiovascular disease; TTE, transthoracic echocardiogram; TOE, transoesophageal echocardiogram; MRI, medical resonance imaging; RBC, red blood cell.

The cardiocoagulum table informs on the potential for genomic domains to weight pathology or image data, evaluate the current clinical significance, or report on the predictive health value. The input of cardiocoagulum quality data will improve precision care intercepts for cardiovascular and hematological-related disorders. P of GIPES, input pathology, and phenomics informatics as life science integrates image-proteomics for multiple medical purposes (Table 1).

Both environment and symptomatic datasets [GIPES] will enable a differential diagnosis in nurture vs. nature and evaluate other factors that personally impact that citizen (Bates et al., 2021). Environment inputs scope multiple causations that include virus to CO2 emissions as the centralization of personalized care cross multiple determinants (Luengo-Oroz et al., 2020; Tan et al., 2020).

Genomic systems embed in healthcare as IPES eXaminations seek to resolve wellbeing and stop high stakes decisions with inherent characteristics that manage populace health as phenotype (Rudin, 2019). Models drive scalable, accurate medicine *via* health records as datasets feature with fast healthcare interoperability resources to abstract informatics and harmonize predictions and precisions with workplace intelligence (Rajkomar et al., 2018).

### Workplace intelligence

Workplace machines and deeply learned intelligence use omics features that target each case with supervised or unsupervised algorithms that learn to abstract value, whilst neural networks activate greater biology insight, such as drug discovery (LeCun et al., 2015). For example, a basic machine-learned algorithm illustrated transitions for high dimensional space as teams progress on linear regression, decision tree and support vector analytics (Hastie et al., 2004, Table 2).

**Table 2:** Basic algorithm and method complexity.

***[Basic Algorithm] A Deep Science Machine—Basic Model Performance Algorithm***	
Feature transformations are followed by a feature selection step and hyperparameter tuning. ML calculates the arguments of the maxima. Given a set of features (F), a target vector (y) and an ML algorithm (M); the performance of the model is given- PM (F, y). Transformation functions as a s̱equence of transformation, (s̱). The objective is to find a set of sequence transformations (S) to produce FNEW = F+S where F ⊂ F satisfies argmax PM (FNEW, y)/S. Meanwhile, the domain of a hyperparameter (N) can be binary, categorial, real-valued, or integer-valued or categorical. Transformations require training and verification of differing model performances.	
*[Method Complexity]*	Y = beta of the intercept(s)
	Random Forest [multiple decision trees]
	Increase vector dimension [multiple dimensions]
Hastie et al., 2004	

“Toward automating data science,” algorithm hyperparameter tuning, progressive sampling, and autonomous assembling better learning using data sets to predict wellbeing (Kanter and Veeramachaneni, 2015; Wistuba et al., 2017). Auto-hyperparameter selection and Bayesian neural science probability inference systems improve Genome-Wide Association studies for predictive value (Feurer and Hutter, 2019; Trochet et al., 2019). As abstract training advanced to Neural Architecture Searches (Table 3), the Welcome Trust supports assurances for the right eXaminations to manage population health (Ordish and Hall, 2020).

**Table 3:** Workplace algorithms.

**Workplace algorithms**				
**Expand and reduce**	**Co-operative Enterprises**	**User-able pipeline**	**Auto-machine LW**	**N.A.S. streams**
AutoLean	Feature tools	TPOT	Frankensteining	Neuro-evolutionary
One button	Feature hub	Auto-WEKA	ML-plan	Tree searching
Explorekit	Git hub	Autosklearn	AlphaD3M	Reinforcement learn

**Figure 2:**
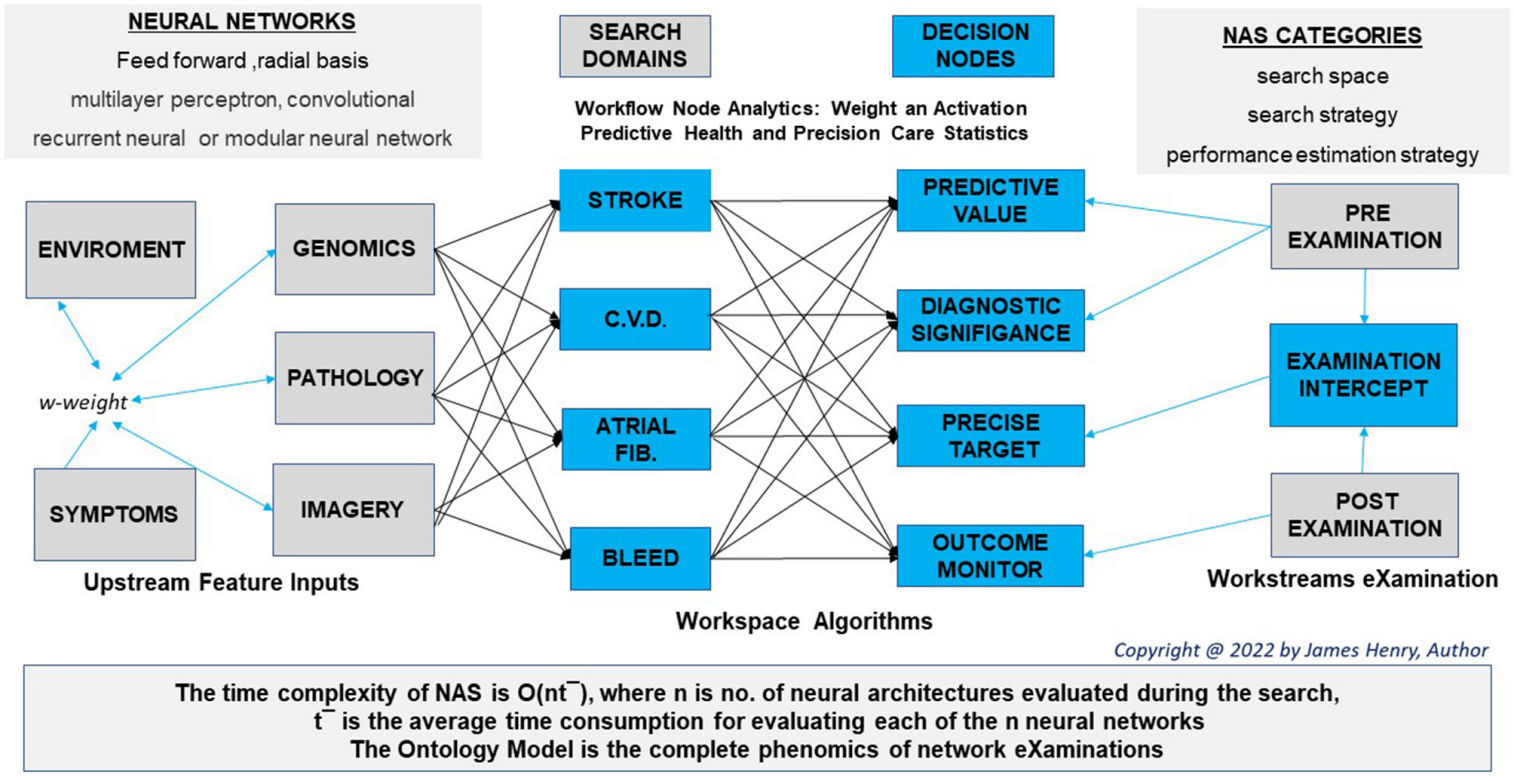
Workflow structure.

Open-source access to basic algorithmic coding scripts contrasts the metadata delivery to multiple NAS networks with individuals and their interactions, developing patterns of knowledge in future health (Ciancarini et al., 2020). Today, NAS dives deep into GIPES data, with intercept precision for Atrial Fibrillation induced Venous Thromboembolism and predictive insight on stroke incidences. In addition, back-propagation from pre-and post-eXaminations better the model ontology for hospital model gains and socioeconomic success (Figure 2).

NAS requirements for model breadth and depth scope assist with a differential and forewarn on a prognosis as a series of weighted abstractions activate predictive health in chronic cardiovascular disease (Lu and Uddin, 2021). Workplace intelligence has time and dimension in a digital workspace dataset for use cases has “voices of biotech research” forecast greater insight to come (Annabi et al., 2021).

### Workstream eXaminations

Diagnostics feature in more than 70% of medical interventions but often fail to deliver informed phenotypes or the most appropriate intervention, whilst future workstreams deliver better citizen health as phenomics datasets integrate use cases to personalize eXaminations (Hallworth, 2011).

Consider the following eXaminations workstream evidence on non-manual clinical support within personalized medicine. The workplace streams insight on diagnostic significance, predictive health, and precision care whilst observing the eXamination intercept. A 44 year old male committed his DNA to a genomic screen and presented to A/E with mild chest pain and palpitations. An electrocardiograph and bloods for analysis were scheduled:

**1st STREAM: Diagnostic significance** included ECG with a short QT interval. Hb 11.1, Platelets 148, White Cell Count 2.9, Chol. 6.1 mmol/l, HDL 0.9 mmol/l, equivocal Trop I. and a viscoelastic raised MCF. A mild angina history scheduled an angiogram.
**2nd STREAM: Predictive health** included sudden death syndrome, with scores on stroke, cardiovascular disease, and bleed risks [Anti-coagulant requirement]. Variants included LDLR and KCNQ- rs2074238 T-allele, F.V. [GWAS coagulative] and V.W.F. [non-pathogenic]. Genome differential eXaminations confirmed an acquired cytopenia [GWAS normal].
**3rd STREAM: Precision care** searched for an ablation, rejected on specialist archive outcomes. Amiodarone, Warfarin, Aspirin, and Clopidogrel were unsupported by VKORC1 and CYPC2C19 polymorphs. Pharmacogenomics targeted an Amiodarone and Rivaroxaban [ABCBI stable] therapy, based on using case data on patient outcomes.
**4th STREAM: Post eXamination outcome** informed on angiogram as non-occlusive with medicinal efficacy monitored by ECG, viscoelasticity, and blood work. Actions where discontinue Amiodarone for Flecainide, while the DOAC dose increased. Mild cytopenia had resolved. All streams presented can develop calculations on uncertainty probabilities with a post eXamination back propagation methodology development.

## Workflow culture

Theorists, philosophers, and psychologists review the bio of logic to conclude on assured AI that best personalize medicine, wherein society does not demark the abstract from the manual decision in health (Englebretsen, 2019). Culture on AI ethics has government recommend digital evaluations for care solutions (Gov.UK, 2020). The NHS actions a people promise to realize workforce alignment (NHS England, 2020b), while AI interprets PHM within eXaminations.

Partnerships with abstract leverage engage communes or providers in forums as system quality groups manage operations to target population health intervention, similar pandemic responses to personalize an intercept (Syrowatka et al., 2021). To culture on biological interventions, an eXaminations best practice readies data engineer and scientist with mathematics and expert clinical and quality practitioner in a workforce workflow to deliver solutions together (Li et al., 2020).

**Figure 3:**
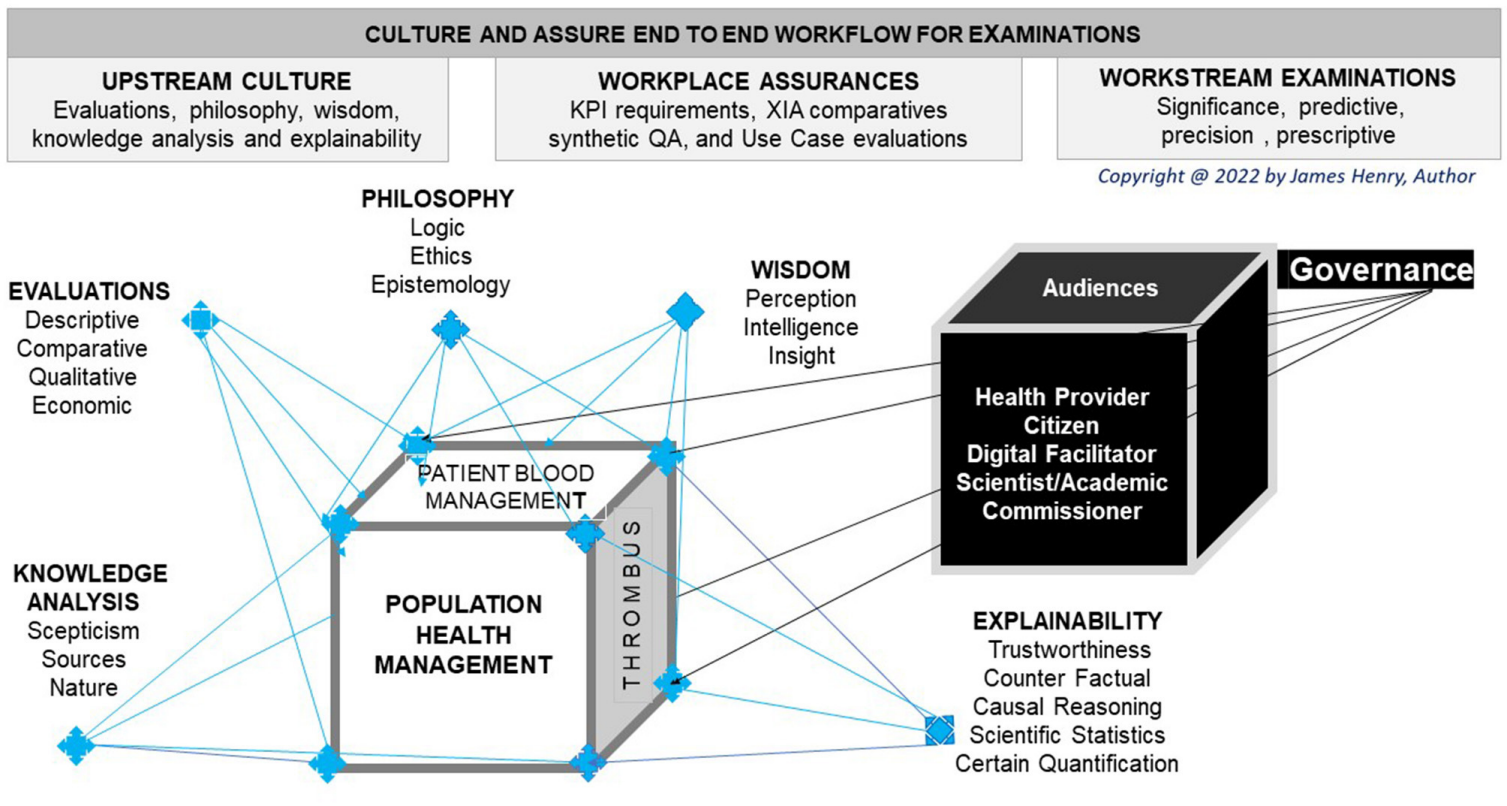
Culture work, assure flow.

The author proposes workflow culture and illustrates multiple groups and arguments to amalgam differing intelligence perceptions that explain AI workplaces and elucidate workstreams to enable PHM (Figure 3).

### Abstract perceptions

Abstract perceptions have academics explain Black Box algorithm incomprehension as beyond a non-scientific analyst's cognitive ability (Nicholson, 2015). Sectors may seek transparency even if an algorithm is not apparent, while observed outcomes inform on lessons to be learned in best practice (Larsson and Heintz, 2020). Positive AI perceptions requires assurances that gain wisdom with a secure digital flow that cultures safe wellbeing in future work to be done (Figure 3).

Like other global efforts, personalized medicine unifies a planet to percept AI benefits (Topol and Lee, 2019). Ownership of data use cases offers many opinions as genomic associations match communities who relate transparency with a concept of knowing (Panch et al., 2019; Larsson and Heintz, 2020). Simplification of transparent intelligence with surrogate learning assists on positive precepts by reason that there are checks on the algorithm to assure the decision (Ferreira and Monteiro, 2020). Transparent integrated care systems align opaqueness with workflow plans for eXamination insight to action change (Table 4).

**Table 4:** Opaque workflow.

**Opaque workflow and transparent plans**			
**Consider access and opaqueness type and the controls required**			**Action plan**
No access	Interpretable Comprehendible Explainable	Opaque Intent	Assess AI framework Validate Assure Verify Synthetic data Track, observe Transacts Insight Action change
Single access	Non interpretable Incomprehensible Unexplainable	Opaque Character	
Dual users	User 1: Non interpretable User 2: Interpretable	User one is less able and perceives opaqueness	
Dual streams	Algorithm 1: Non interpretable Algorithm 2: Interpretable	Algorithm 1: presents as opaque universally	

### Explicable workplace

As a society, we evaluate biological truth and present evaluations, philosophy, wisdom, knowledge analysis, and explainability (Figure 3). Robust associations in system biology provide research eXaminations for phenomics integration, whence collaborated upon, and governed appropriately (Uffelmann et al., 2021). The explicable workplace advances the approaches and challenges of explainable abstracts to be commissioned for our future health (Xu et al., 2019).

One foresees conventional scientific determinism with abstraction explaining the biological models through PHM (Veatch, 1970). Therein coordinators develop explicable workplaces with interpretable workflow eXaminations that instill trust with operations from cardio epigenomics to coagulum protein modification (de Vries et al., 2020; Schiano et al., 2020). While scientific explanation remains debated, AI surpassed clinical performance over several genomic domains with greater success envisaged in healthcare to improve the citizen lifecycle journey (Veatch, 1970; Fogel and Kvedar, 2018).

Nevertheless, consignment of upstream datasets to format feature use cases brings global ethical queries to workstream eXaminations since they support proportional or definitive prognosis to specify human biology and precision therapy as a decision-making process (Jobin et al., 2019). Responsible, fair and justified ethical principles for genomic informatics foresee the phenotypes while other AI abstracts aim for precision insights to personalize pre ontology intercepts that individualize an eXamination as an implementation plan to improve outcomes (Jobin et al., 2019).

Trust shifts from heuristics to abstracts when monitoring patient outcomes with AI evidence, similar to oncology therapy and their post eXamination follow up with robust scientific support (Kimmelman and Tannock, 2018). A user reasons the counterfactual in assessing needs to speed up requisition of eXaminations that reduce the frequency or severity of harm; therein consider precision medicine without sequence analysis to reason health is not affected by DNA variation, to argue for pharmacogenomic evaluation and safe wellbeing (Prosperi et al., 2020).

Trustworthy claims about algorithms and those made by an algorithm reason an appropriate use case that does not require law, except in claims that AI is trustworthy when not assured, insured, elucidated or agreed upon (Spiegelhalter, 2020).

### Elucidate workstreams

Explaining an ontology or hospital workstream that reduces mortality rates requires elucidation of the eXaminations with checks on any bias to enable a test interpretation and a valid decision. AI users more readily accept the abstract process for a support decision when performance indicators, surrogate AI, synthetic QA, and commissioned use cases become the norm (Figure 3).

Workstream assurances offer a “judgement, making decisions and prediction,” while mitigating adversity with model-specific or agnostic methods and synthetic data (Ahmed et al., 2020; Chen et al., 2021). Specific tools are intrinsically interpretable, belonging to the essential nature of an algorithm with visibility into how an AI system makes decisions, with the standard deviation founding a “Glass Box (Rai, 2019). Agnostics methods elucidate the black box with AI explanations, with examples for partial dependence and causal interpretation, individual conditional expectation, and accumulated local effects (Renki, 2020).

Neural network practice in medicine futures elucidate data in biotechnology, for a biological or therapeutic specification, as AI presents accurately what the clinician cannot (Sanal et al., 2019). Elucidating work through data mining or in images from place to stream requires assessment and classifiers before extracting knowledge with machine and deep learning (Vidushi Agarwal and Rajoria, 2019; Aggarwal et al., 2021). Communities and health providers engage enhanced computing power and cloud storage to mitigate bias and improve decision accuracy with improvements in workflow that converge the workforce (Topol, 2019). Healthcare models dispel AI apprehension and revolutionize wellbeing with an ICS PHM that brings a norm to organizations and personnel impact (Spatharou et al., 2020).

Consider the term artificial, which invokes a query on machine-made that requires an explanation and elucidation of the stream for positive perception. The word intelligence seeks clarity in a workplace definition as ML and DL use case algorithms abstract upstream data for workstream eXaminations that culture flow and assure work (Figure 3).

## Step by step design

**Figure 4:**
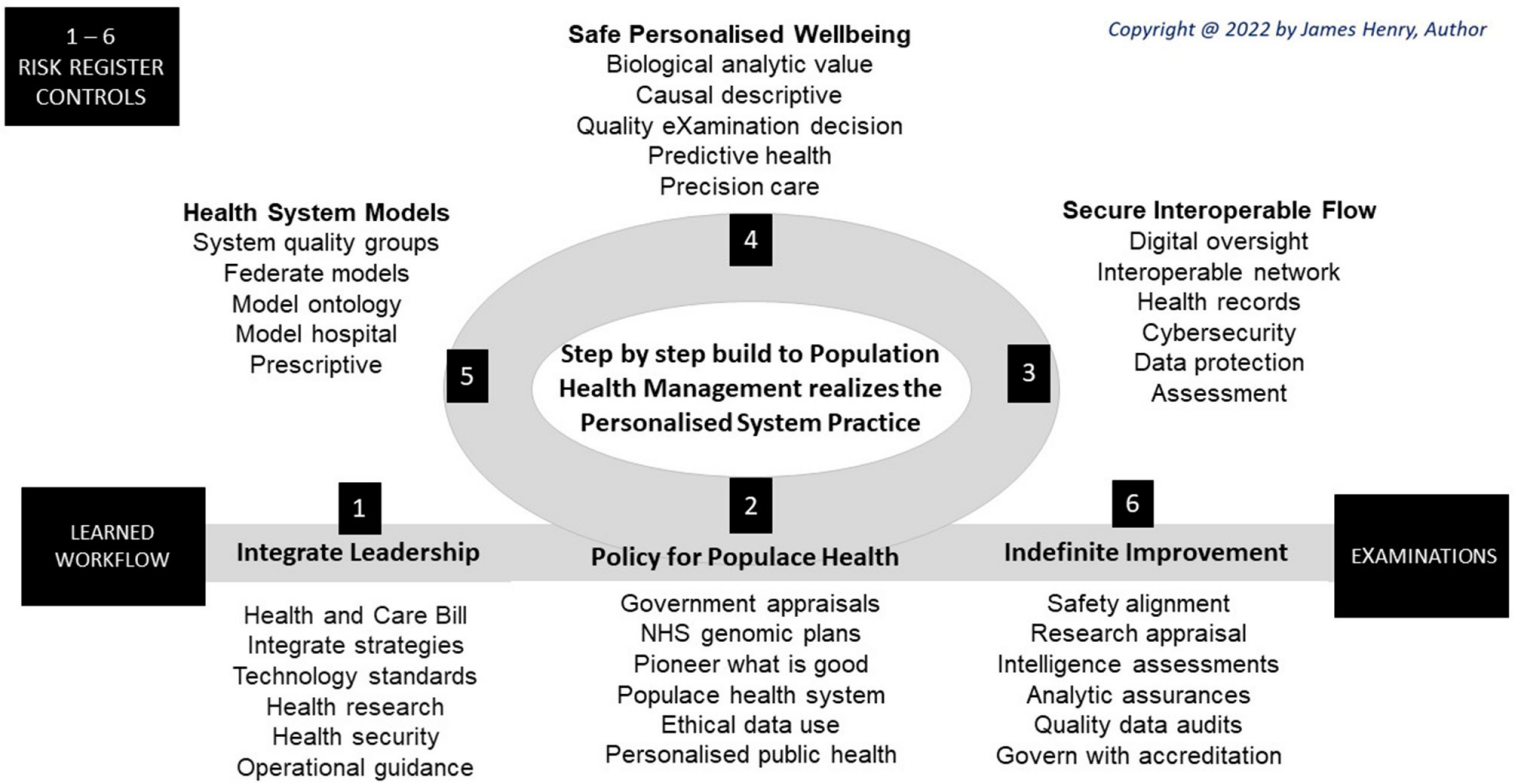
Workflow steps.

The NHS builds on a culture for insight normality by enabling step-by-step processes to realize the digital, data, and technology vision in a long-term NHS plan (Department of Health and Social Care, 2018; GOV.UK, 2019a). Bringing society together with recommendations from the Francis, Carter, and Paterson reports (House of Commons, 2013, 2020; Department of Health, 2016) require key step enablers to mitigate variance or adversity, as shown (Figure 4).

### Step 1. Integrate leadership

In 2022, the UK Health and Care Bill enactment brings rigidity to steps designed for NHS recovery with cohesive and safer services to tackle long-term waiting lists, chronic conditions, and health inequalities^1^ A global genomic lead has national strategies for innovation, AI, data, and commission of a core purposes to improve outcomes, tackle inequalities, and enable productivity with socioeconomic development (GOV.UK, 2019b, 2021a,b; Global Alliance for Genomics and Health, 2021; NICE, 2021; NHS, 2022b). Thereby future wellbeing has integrated system governance of steps for an eXamination norm (NHS, 2022b, Figure 4).

To unite eXaminations, a UK code of practice assists the government in the design and build of technology to deliver secure and ethical workflow functions (GOV.UK, 2021c). At the same time, an NHS standard reinforces best practices (NHS, 2021a). Our future health programme underpins public health eXaminations in developing new ways to prevent, detect and treat disease (NHS, 2020). The Health Research Authority and the Biomedical Catalyst Program assist researchers and businesses develop projects that personalize an efficacious pre- to post-eXamination process that also assures the intercept (UK Bioindustry Programme, 2020; NHS Health Research Authority, 2021).

ICS leadership focus on the patient's perspectives with accountability for safe digital delivery as clinical and life science experts with technology partners assess citizen and health provider needs for the commission of the workflow eXamination (NHS, 2022b). After that, the UK Health Security Agency and the Healthcare Safety Investigation Branch can protect the community from the impact of health threats and provide intellectual, scientific, and operational leadership for secure wellbeing that can review an eXamination process and its system management (GOV.UK, 2021d; Healthcare Safety Investigation Branch, 2021).

### Step 2. Plan population health

Integrated care with a system for the management of populace health requires “approved thinking models” with personalized practices to underpin advice that clarifies socioeconomic, NHS and public benefits in the delivery of policy aims (NHS, 2021b). NHS England plan with Genome UK on implementation commitments that action pillars to predict health and precision care (GOV.UK, 2006).

Digital electrical and electronic engineers pioneered autonomous models for human-centric aims, ethics, and value, with mitigation of privacy and security risk (IEEE Advancing Technology for Humanity, 2017). Work for what good looks like frame digital principles to describe how ICS arrangements support success as maturity assessments level up and deliver population management *via* health records (NHS, 2021c).

Principal steps transition to analytic norms in populace health as digital genomics develop health care system eXams built on secure interoperable flow for safe personalized wellbeing (Figure 4). Model ontology and model hospitals develop with plans for Microsoft Healthcare and Genomic Laboratory Information Systems (Microsoft Industry, 2015; Digital Health, 2021a).

Personalized public health requires the integration of research innovations with ethics for “appropriate and responsible data use in government and the wider public sector” (Government Digital Service, 2020). Appropriate public policy projects foresee the genomic revolution and plan digital health for data research with respect for the person, genetic solidarity, and altruism (Public Policy Projects Public Policy Institute, 2021).

### Step 3. Secure interoperable flow

The NHSE transformation directorate oversee digital strategy and policy with project commissions for secure technologies and open standards to meet populace needs on public health platforms (Digital Health, 2021b; NHS Digital, 2022a). Systems support framework support populace health to integrate care with networks to analytic suppliers with scope limited to non-clinical services (NHS England, 2022).

NHSE digital aims accelerate partner working to make effective resource use, with scope for virtual wards, digitization, and skills (NHS England, 2022). Interoperable access to secure flow in personalized health has API and FHIR enabling populace wellbeing (Alterovitz et al., 2020), as mobile app evaluation, education, and awareness of digital formularies better health (Lagan et al., 2020).

A nationwide e-health record enables populace health management with system links to data from multiple settings which enable biological ontology workflow (Wood et al., 2021). Data mining records enables better research for applications in clinical care, as cardiocoagulum learning on genetic data to phenotype depend on secure interoperable flow (Jensen et al., 2012; Zhao et al., 2019).

NHSE Transformation Directorate oversees security with the “WannaCry” crisis, a wake up for a cyber-service essential framework for process review and response to alerts in toolkits (Hoeksma, 2017; NHS Digital, 2022b). DCB standards issued by NHSE Transformation Directorate require IT system manufacturers and healthcare organizations to carry out a product risk assessment to determine acceptance (NHS Digital, 2022c).

Digital Technology Assessment Criteria for secure interoperable flow instills provider and community confidence that access, usability, interoperability, and technical security meet assurances (NHSE Transformation Directorate, 2021). Both data protection and clinical safety processes require a sign off evaluation process in transition to safe populace wellbeing, as itemized (Table 5).

**Table 5:** Data protection and clinical safety.

**Criteria**	**Officer**	**ICB/ICP**	**Safety**	**Conform**	**Oversight**
Data protection	Information commissioner	Protect personal data [consent]	Registration self-assess	Data protection act 2018	ICO
	Data protection	Protect workflow data	Data protection policy	Data protection act 2018	ICO
Clinical safety	Provider clinical safety	Maintain health IT system	Clinically risk manage	DCB0129 DCB 0160	NHS digital
	MHRA enforcement	Maintain medical devices^∧^	Medical Device requirement	UK MDR 2002	MHRA UK MDR 2002 ^∧^

^∧^Public Consultation.

MDR, medical device regulations; ICO, Information commissioner's office.

### Step 4. Safe populace wellbeing

**Figure 5:**
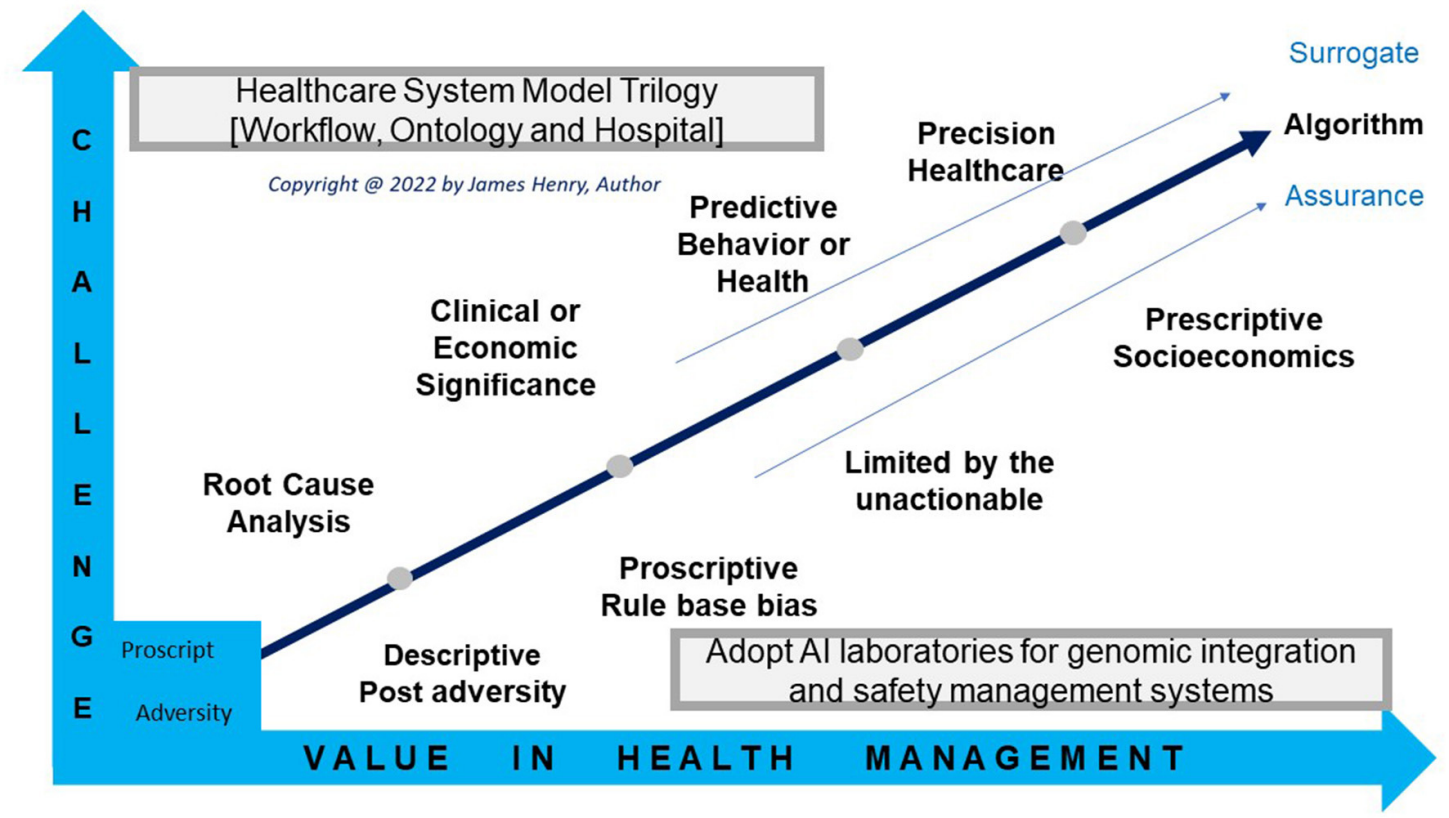
Workflow values.

Society seeks explanation to present significance and describe incidence as clinically relevant at reviews to integrate GIPES insight to manage populace health. Big data analytic challenges offer biological value for safe populace wellbeing, social behaviors, and economics with descriptive, predictive, precision, and prescriptive points of interest, so mapped (Figure 5).

Critical realists of the causal descriptive use eXaminations to present a pathology or explain adverse outcomes which inform on a phenotype variation or a decision bias (Fox and Aranko, 2017). Building eXaminations from GIPES descriptive data to foresee the clinical significance, morbidity, or mortality can resource predictors before the event which requires data mining of electronic health records (Tomašev et al., 2021).

Quality decisions from pathology results, input clean “examination” data as a norm to manage diagnosis, while GIPES feature in AI use cases for biological “eXaminations” that personalize health [International Organization for Standardization (ISO), 2017; Get It Right First Time, 2021; ISO/DI 15189 [Internet], 2022]. AI builds across places after experts simulate health efficacy or hospital efficiency, wherein data scientists validate eXaminations to commission and accredit excellence for health providers to adopt the integrated system [ISO/DI 15189 [Internet], 2022; NHS, 2022b].

AI laboratory eXaminations predict pathology to personalize intercepts in models when assessed on the validity of the process in the future of healthcare (Barker, 2017; de Hond et al., 2022). Advances in predictive health are restricted in the volume and veracity of GIPES data as analytics trial patterns in associations of predictors in VTE outcomes, like A-Fib polygenic risk scores with; monogenic variables (Abraham et al., 2021), clinical risks (O'Sullivan et al., 2021), and lifestyle choice (Ye et al., 2021).

Precision terms offer Pre-eXamination value to intercept as the data mining of GIPES informatics embrace more than genomic alone to define or insight phenotype with predictor searches to exact an interventional choice (Abraham et al., 2021; O'Sullivan et al., 2021; Ye et al., 2021), as an eXamination norm. For instance, VTE predictive power can precision intercepts similar pharmaceuticals, lifestyle choices and invasive procedures, such as A-Fib ablation (Burstein et al., 2017; Mai et al., 2019; Kolin et al., 2021; Yuan et al., 2021).

### Step 5. Healthcare system models

Models manage disease predictor or business opportunity and develop genomic systems with operations for cloud computing and AI laboratories [graph A]. As people live longer with co-morbidities society address health system fragmentation with strategies to execute models (GOV.UK, 2019b, 2021a,b; Global Alliance for Genomics and Health, 2021; NICE, 2021; NHS, 2022b). Thereby a system quality group and a local needs quality group terms of reference abstract decision-making for ontology and hospital gains with partners sharing system insights that predict health and precision care (National Quality Board, 2022a).

Federating datasets enables digital solutions to abstract outcome with accurate and robust statistical health system models (Rieke et al., 2020). Population health management improve quality of care and reduce cost growth with model ontology and hospital (Berwick et al., 2018). The Systematized Nomenclature of MEDicine -clinical terms provides a vocabulary norm for e-informatics exchanges wherein ontology prediction and precision care are realized. Model hospitals plan with social care to lower burdens with business intelligence supported by SNOMED (NHS Digital, 2018).

Learning communities expand on biomedical space to GIPES dimensions to improve wellbeing and social system challenges as federated models provide a rational on ontology and hospital eXamination solutions for socioeconomic success (Hill, 1984; Xu et al., 2020). Descriptive to predictive repositories add post eXamination intercept data to enable neural network to back propagate on models and prescript control by cognitive analysis (Hennessy et al., 2015; Whittington and Bogacz, 2019).

### Step 6. Indefinite improvement

**Figure 6:**
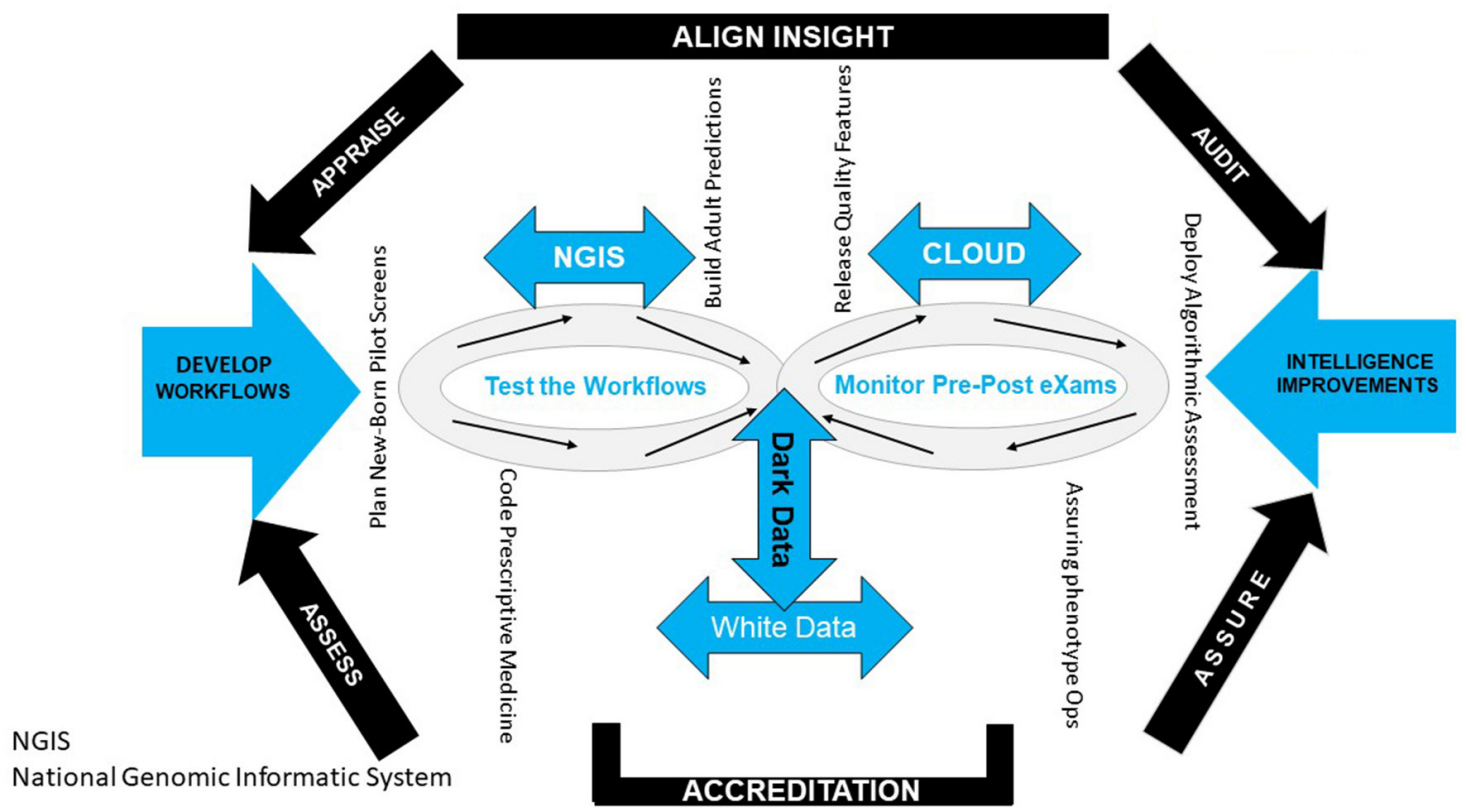
Workflow governance.

Data administrators, engineers, and analysts ingest, process, and visualize better health and social care whilst digital improvement becomes a legal requirement of ICS organizations for a license to operate (Carding, 2022). To govern personalized care, then six A's, align, appraise, assess, assure, audit, and accredit healthcare eXaminations. Workforce healthcare management imputes a workflow bot for national improvements executed regionally for safe health and social care delivered locally (Figure 6).

The Health Research Authority action health and social care with portfolio appraisals to put people first for indefinite improvement as workflows with GIPES databases (NHS Health Research Authority, 2021; NHS, 2022c). The Academic Health and Science Networks assess community and health provider needs for AI laboratory developments, similar A-Fib projects, and community pharmacies to support cardiocoagulum proposals (The AHS Network, 2020; Turner et al., 2020).

Recommendations in the Francis, Paterson and Carter reports explaining, in part, the approximation of 11 K deaths from patient safety incidents each year (House of Commons, 2013, 2020; Hogan et al., 2015; Department of Health, 2016). To assure our future health and reduce elective waiting lists, the ontology and hospital eXaminations accumulate descriptive to predictive data to precision and prescript wellbeing near to patients (Our Future Health, 2021; Get It Right First Time, 2022).

The Medical Healthcare and products Regulatory Authority audits clinical trial conduct whilst the Care Quality Commission re-focus on auditing information and fewer on-site inspections (Khin et al., 2021; Care Quality Commission, 2022). The Data Alliance Partnership Board enable data collections whilst a Records Management Code of Practice audits contracted organizations, adult social care, and public health on standard data migration (NHS, 2021d; NHS Digital, 2022d).

Accrediting workflow workstream eXaminations originates in a quality report from 2014 (Henry, 2017). A shared national quality policy actions internal and external, qualitative, and quantitative functions (Burstein et al., 2017; National Quality Board, 2022b). The National Institute of Health and Clinical Excellence and Health Education England develop standards and AI programme training (NICE, 2021; NHS Health Education England, 2022). International standards enable governance of data use cases with an eXamination process control and a safe AI risk management system that personalizes practice [International Organization for Standardization (ISO), 2017, 2018b; ISO, 2022; UKAS, 2022].

Taken collectively the 6 A's—align, appraise, assess, assure, audit, and accredit- personalized care as an evidence unit as the system workforce demonstrates a citizen centric impact. By sharing decision making between clinicians and the public whilst utilizing intelligent abstracts in future health provides greater value with predictive health and precision care with realistic expectation and a therapy choice that offers more appropriate interventions on wellbeing^2^.

## Summary

Integrating hospital with social care require a personalized end-to-end structure for intelligence eXaminations that support decisions. Oversight by system quality groups embeds a positive culture for abstract explanation and elucidation with context to transparent workflow. Such support to workforces brings confidence to plan and perform data functions upstream that place personal use cases in the workstream.

Digital health laboratories work for communities with stakeholders and partner co-operations to accelerate Population Health Management to personalize care. Senior Programme Management's commitment to public safety develops effective plans for healthcare systems that expedite digital eXaminations for wellbeing, regardless of the place of access.

Personalized care groups with integrated organizational-wide functions require technology to scale populace health with knowledge accumulation and best-practice analytics for agile service. Health service governance aims for a program to benefit personalization, working with teams on long-term plans to execute innovation, digital, data, and excellence pillars as genomic commitments better future health.

Getting personalized care right the first time requires informatic resilience and coding scripts that place the citizen centric. In future health, population health management enhances public outcomes and impacts productivity. Corporate programme managers plan model ontology with model hospital and action workflow functions to enable practitioner reviews that confirm effectiveness and efficiencies nationally, regionally, and locally.

Strategy development with analytics operates business intelligence into our future health. It links hospitals with social care to identify socioeconomic burdens and deliver personalized care group oversight on personalized health and care programme solutions. Change control readies with NHSE Directorates, personalized care groups, and stakeholder support of health industry controls that evidence cases.

## Conclusion

Global apportioning of health-related informatics readies for predictive health and precision care. National challenges raised by partner and provider to integrate genomic care requires deep understanding of intelligent workflow for the management of populace health. Digital data-driven non-manual outputs support personalized medicine decisions regarding a bleed or thrombus event. Workflow delivers citizen system biology to general practice, Hospital Trust, and community as digital AI develop phenotype eXaminations.

Upstream, workplace and workstream end-to-end solutions feature genomics, images and pathology data, where AI abstracts develop better admin and clinical operative decisions that increase productivity and enhance health outcomes. Culturing abstract algorithms has workforce share perceptions with system quality groups joint, working committees and communes by engagements that elucidate workstreams in a personalized practice proposal.

Workflow steps integrate leadership to plan health management policies with insights from transformation and improvement actions of an eXamination process. Steps secure the digital flow of data for safe wellbeing and observe health service practice with safety insight for an NHS recovery. Culture intelligent workflow, structure, culture, and steps proposes leading groups and improving personalized practice for national opportunities, regional priorities, and local needs. Aligning organization, practitioner, genomic and social variations by overseeing descriptive, predictive, precision, and prescriptive analytics commences by culturing the step-by-step design for population health management.

## Author contributions

The author confirms being the sole contributor of this work and has approved it for publication.

## Footnotes

^1^Health and Care Bill—Parliamentary Bills—UK Parliament [Internet]. Available online at: https://bills.parliament.uk/bills/3022.

^2^14:00-17:00. International Organization for Standardisation (2020).
